# Effects of Different Levels of *Lycium barbarum* Flavonoids on Growth Performance, Immunity, Intestinal Barrier and Antioxidant Capacity of Meat Ducks

**DOI:** 10.3390/antiox14010067

**Published:** 2025-01-08

**Authors:** Minhang Tu, Gentan Cai, Longfei Ma, Leyan Yan, Tian Wang, Zhendan Shi, Chao Wang, Zhe Chen

**Affiliations:** 1 College of Animal Sciences and Technology, Nanjing Agricultural University, Nanjing 210095, China; tuminhang@stu.njau.edu.cn (M.T.); caigentan@stu.njau.edu.cn (G.C.); malongfei@stu.njau.edu.cn (L.M.); wangtian@163.com (T.W.); 2 Institute of Animal Science, Jiangsu Academy of Agricultural Sciences, Nanjing 210014, China; yanleyan198469@126.com (L.Y.); zdshi@jaas.ac.cn (Z.S.); 3 Integrated Crop-Livestock Systems Key Laboratory, Ministry of Agriculture and Rural Affairs, Nanjing 210014, China

**Keywords:** *Lycium barbarum* flavonoids, meat duck, growth performance, intestinal function, antioxidation

## Abstract

**Background:** In vitro findings on the biological functions of Lycium barbarum flavonoids (LBFs) as feed additives are limited. This study aimed to explore the effects of different concentrations of LBFs on the growth performance, immune function, intestinal barrier, and antioxidant capacity of meat ducks. A total of 240 one-day-old male meat ducks were randomly allocated to four groups, each receiving a basal diet supplemented with 0 (control), 250, 500, or 1000 mg/kg of LBFs for 42 d. **Results:** The results showed that dietary supplementation with 500 mg/kg of LBFs resulted in a significant increase in average daily feed intake, body weight, average daily gain, and feed conversion ratio. Dietary supplementation with 500 or 1000 mg/kg of LBFs resulted in significant decreases in serum levels of D-lactic acid and lipopolysaccharide. Dietary supplementation with 500 mg/kg LBFs significantly decreased diamine oxidase activity and enhanced the activities of catalase, total antioxidant capacity, and glutathione peroxidase in the jejunal mucosa, as well as the activity of total superoxide dismutase and the content of glutathione in the ileal mucosa, while significantly lowering the content of malondialdehyde in the ileal mucosa. Dietary supplementation with 500 mg/kg LBFs significantly up-regulated the mRNA expression of genes associated with intestinal barrier function and antioxidant capacity in the jejunal and ileal mucosa, as well as the protein expression of these antioxidant genes, and led to a significant reduction in the mRNA expression of pro-apoptotic and inflammatory-related genes. **Conclusions:** The addition of LBFs to the diet improved the growth performance, intestinal barrier function, immune response, and antioxidant capacity of the ducks, which may be closely associated with the activation of the Nrf2 signaling pathway and the inhibition of the NF-κB signaling pathway. The optimal dietary inclusion level of LBFs in ducks was 500 mg/kg.

## 1. Introduction

The duck meat industry has significantly contributed to animal protein consumption, human nutrition, and global food security [1]. As the industry evolves toward production systems that are more intensive and commercialized than those often used today, an increasing number of adverse factors are affecting the health and growth of ducks, including deterioration of rearing conditions, increased stocking densities, heat stress, and oxidative stress [2,3]. Such negative influences reduce growth performance and meat quality and increase mortality rates in sheep and broilers, leading to a substantial waste of farming resources and considerable economic losses for producers [4]. Similar effects may also be observed in ducks. The gut of meat ducks plays a pivotal role in the regulation of nutrient absorption and is crucial for immune and stress modulation. Enhancing gut health optimizes nutrient distribution and supports organ development, tissue growth, and maturation of the immune system [5,6]. Plant extracts, known for their diverse biological functions, high efficacy, and safety, can be incorporated into diets to enhance gut morphology, mucosal barrier integrity, immune function, and antioxidant capacity, promoting healthy growth and development of meat ducks [7,8].

Flavonoids are efficient natural antioxidants that protect tissues from oxidative damage by scavenging free radicals and inhibiting lipid peroxidation. Goji berries (Lycium barbarum, LB), a traditional Chinese herbal medicine, have been widely used as raw materials in functional foods for centuries [9,10]. Flavonoids extracted from LB are referred to as Lycium barbarum flavonoids (LBFs), which are marketed as a yellow-brown crystalline powder and are considered the primary active components responsible for its antioxidant activity [11,12]. Studies in mice have shown that LBFs can improve antioxidant function by enhancing serum antioxidant enzyme activity and reducing lipid peroxide levels and increase antioxidant capacity by elevating the free radical-scavenging rate in macrophages [13].

Despite promising in vitro findings regarding the multiple biological functions of LBFs, including their antioxidant, anti-inflammatory, and immunostimulatory effects, [13,14], research on their use as feed additives is limited. Therefore, in this study, we aimed to explore the effects and optimal dosage of LBFs in meat ducks through dietary supplementation and investigate their mechanisms of action. The research will assess any theoretical support for the practical application of LBFs in the meat duck industry.

## 2. Material and Methods

### 2.1. Animal Care

The Nanjing Agricultural University Institutional Animal Care and Use Committee approved the animal testing and associated protocols used in this work (permit number: SYXK-2020-00176).

### 2.2. Experimental Design and Animal Management

In this study, a total of 240 1-day-old Cherry Valley ducks were sourced from a local hatchery and randomly allocated to four treatment groups (6 replicates per group, with 10 birds per replicate), receiving experimental diets supplemented with 0 mg/kg (control), 250 mg/kg, 500 mg/kg, and 1000 mg/kg of LBFs added to the basal diet over a 42-day experimental period. All meat ducks were housed in multi-tiered cages of equal height, with each cage accommodating ten ducks and lined with plastic mesh flooring of 2.0 to 2.2 cm mesh size. During the experiment, ducks were provided with starter diets from days 1 to 21 and then switched to grower diets from day 22 until the end of the 42-day trial. The basal diets met or exceeded the nutritional requirements established by the National Research Council (NRC, 2012), with the corresponding ingredient compositions and nutritional levels detailed in Table 1. For the first three days, the indoor temperature was kept at 34 ± 1 °C. Thereafter, the temperature in the duck house was gradually decreased by approximately 1.5 °C every three days, reaching a final room temperature of 21 °C by day 21. Indoor relative humidity was maintained at 45–55% throughout the entire experimental period. Body weight (BW) and feed intake of ducks were measured on days 1 (after hatching), 21 (fasted for 12 h), and 42 (fasted for 12 h). Subsequently, average daily feed intake (ADFI), average daily weight gain (ADG), and feed conversion ratio (FCR) were calculated for the developmental stage (days 1–21), growth stage (days 22–42), and the overall experimental period (days 1–42).

### 2.3. Sample Collection

At the end of day 42 of the experiment, six healthy meat ducks were randomly selected from each replication group for weighing. Blood samples were collected from the jugular vein and centrifuged (4 °C, 3000 rpm, 15 min) after 2 h at room temperature to prepare serum; the supernatant was stored in a −80 °C refrigerator for testing. The meat ducks were dissected to isolate the jejunum and ileum. The isolated intestinal segments were gently rinsed with 0.9% sodium chloride (NaCl) solution to remove residual food particles and other contaminants. Using surgical scissors, the jejunum and ileum were carefully opened longitudinally, and the intestinal mucosa was scraped using a sterile glass slide. The jejunum and ileum mucosa samples were homogenized with 0.9% NaCl solution (1:9) and centrifuged (4 °C, 3000 rpm, 15 min), after which the resulting supernatant was collected. The remaining mucosal samples were transferred to cryovials, and both the samples and the supernatant were rapidly frozen in liquid nitrogen before being stored at −80 °C for subsequent analysis. After slaughter, the spleen, thymus, bursa of Fabricius, pectoral muscles, and leg muscles were weighed to calculate the relative weights of immune organs using the following formula: Immune organ index (g/kg) = organ weight (g)/live body weight (kg).

### 2.4. Indices Measured by Enzyme-Linked Immunoassay (ELISA)

The activity of D-lactic acid (D-LA, Catalogue no. MM-91644O1), lipopolysaccharide (LPS Catalogue no. MM-1789O1), and diamine oxidase (DAO, Catalogue no. MM-1789O1) in serum was measured by ELISA with the corresponding kits provided by Nanjing Qiang ke Biotechnology Co., Ltd. (Nanjing, China).

### 2.5. Antioxidative Indices in Jejunum and Ileum

Jejunal and ileal mucosal antioxidant indicators included glutathione (GSH, Catalogue NO.A006-2-1), malondialdehyde (MDA, Catalogue NO.A003-1-2) concentration, total superoxide dismutase (T-SOD, Catalogue NO.A015-2-1), glutathione peroxidase (GSH-Px, Catalogue NO.A005-1-2), catalase (CAT, Catalogue NO.A007-2-1), and total antioxidant capacity (T-AOC, Catalogue NO.A015-2-1) activity assays, which were conducted using assay kits (Nanjing Jian cheng Bioengineering Institute, Nanjing, China). To allow for comparisons between samples, the obtained data were normalized to the total protein content of each sample.

### 2.6. RNA Extraction and Quantitative Real-Time PCR Analysis

Total RNA was extracted from the jejunum and ileum mucosa according to our previous method [15], and the relative expression of mRNA was analyzed. The primer sequences for the target genes related to antioxidant activity (superoxide dismutase1 (*SOD1*), *GSH-Px*, heme oxygenase-1 (*HO-1*), nuclear factor erythroid-2-related factor 2 (*Nrf2*), NAD(P)H dehydrogenase, and quinone1 (*NQO1*)), immunity (nuclear factor kappa-B (*NF-κB*), myeloid differentiation primary response 88 (*MyD88*), Secretory Immunoglobulin A (*SlgA*), interleukin-2 (*IL-2*), tumor necrosis factor-a (*TNF-α*), and interferon-c (*IFN-γ*)), intestinal barrier (Mucin2 (*MUC2*), occludin (*OCLN*), claudin1 (*CLDN1*), claudin2 (*CLDN2*), zonula occludens-1 (*ZO-1*)), and cell proliferation and apoptosis (B-cell lymphoma 2 (*Bcl-2*), Bcl-2-associated X (*BAX*), marker of proliferation Ki-67 (*Ki67*), and cysteinyl aspartate-specific protease 3 (*Caspase3*)) were synthesized by Nanjing Qing ke Biotechnology Co., Ltd. (Nanjing, China). The primer sequences utilized in this experiment are presented in Table 2.

### 2.7. Analysis of Western Blotting

Western blotting analysis of jejunum and ileum mucosa was carried out according to previous studies [16]. The primary antibodies including Nrf2 (1:1500), Kelch-like ECH-associated protein 1 (Keap1) (1:5000), HO-1 (1:8000), and NQO1 (1:4000) were provided by Cell Signaling (Danvers, MA, USA). The secondary antibody (1:5000; Proteintech Group, Inc., San Diego, CA, USA) was horseradish peroxidase-labeled goat anti-rabbit immunoglobulin G in the present study. β-actin was used as the internal reference protein.

### 2.8. Statistical Analysis

The experimental data were subjected to a one-way analysis of variance using SPSS 22.0 software, and the results are expressed as mean ± standard error of the mean (SEM). Multiple comparisons among groups were conducted using Duncan’s multiple range test, while linear and quadratic effects were examined via orthogonal polynomial contrasts. Differences were considered statistically significant at *p* < 0.05.

## 3. Results

### 3.1. Growth Performance

As shown in Table 3, dietary supplementation with 250, 500, and 1000 mg/kg LBFs significantly increased the body weight of meat ducks on day 42 compared with that of the control group (*p* < 0.05). During the developmental phase (1–21 d), dietary supplementation with 250, 500, and 1000 mg/kg LBFs decreased the ADFI (*p* < 0.05). Dietary supplementation with 250 and 1000 mg/kg LBFs also significantly improved the FCR. During the growth phase (22–42 d), dietary supplementation with 500 and 1000 mg/kg LBFs significantly increased the ADG compared with that of the control group (*p* < 0.05). Additionally, 1000 mg/kg LBFs significantly improved FCR. Over the entire experimental period (1–42 d), and using the control group data as the baseline, dietary supplementation with 250, 500, and 1000 mg/kg LBFs significantly increased ADG and improved FCR (*p* < 0.05). Notably, 500 and 1000 mg/kg LBFs also significantly increased ADFI (*p* < 0.05).

### 3.2. Organ Index

As presented in Table 4, dietary supplementation with 250, 500, and 1000 mg/kg LBFs did not significantly affect the relative weights of the spleen, pancreas, bursa of Fabricius, liver, pectoral muscles, or the abdominal fat index of the meat ducks (*p* > 0.05).

### 3.3. Intestinal Tract Permeability

**Figure 1:**
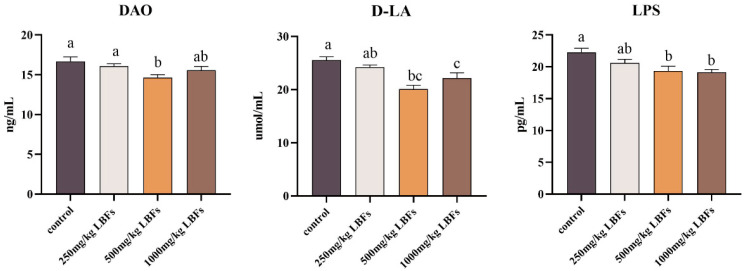
Effects of dietary LBFs on serum intestinal barrier indicators of meat ducks. Note: The mean and SEM are used to present the results, n = 6. Values with different superscript letters are significantly different, as shown by a, b, c (*p* < 0.05).

As shown in Figure 1, serum D-LA and LPS contents and DAO activity in meat ducks decreased linearly with increasing dietary LBF levels (*p*-linear < 0.05). Dietary supplementation with 500 and 1000 mg/kg LBFs significantly reduced serum D-LA and LPS contents, and 500 mg/kg LBFs significantly reduced serum DAO activity (*p* < 0.05).

### 3.4. Antioxidant Status in Intestinal Mucosa

As shown in Table 5 and Table 6, compared with the control group, dietary supplementation with 250, 500, and 1000 mg/kg LBFs significantly increased GSH content in the ileal mucosa (*p* < 0.05); dietary supplementation with 500 and 1000 mg/kg LBFs significantly increased T-AOC activity in the jejunal and ileal mucosa, GSH-Px activity in the jejunal mucosa, and T-SOD activity in the ileal mucosa and significantly decreased MDA content in the ileal mucosa (*p* < 0.05); and dietary supplementation with 500 mg/kg of LBFs significantly increased CAT activity in the jejunal mucosa (*p* < 0.05).

### 3.5. Cell Apoptosis-Related Genes Expression in Intestinal Mucosa

**Figure 2:**
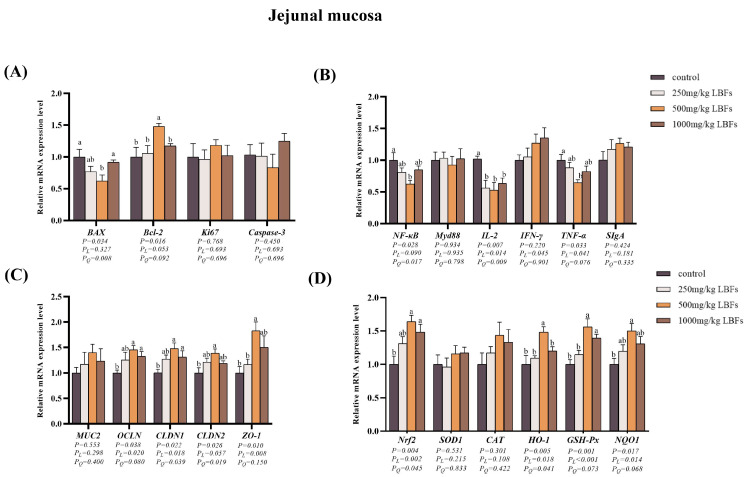
Effects of dietary LBFs on the mRNA expression of proliferation-, apoptosis-, immunity-, intestinal barrier function-, and antioxidant-related genes in the jejunal mucosa. (**A**) Relative mRNA expression levels of proliferation- and apoptosis-related genes. (**B**) Relative mRNA expression levels of immune-related genes. (**C**) Relative mRNA expression levels of intestinal barrier-related genes. (**D**) Relative mRNA expression levels of antioxidant-related genes. Note: The mean and SEM are used to present the results, n = 6. Values with different superscript letters are significantly different, as shown by a, b (*p* < 0.05). *Q* and *L* are the quadratic and linear responses, respectively, to the levels of dietary supplementation with LBFs.

**Figure 3:**
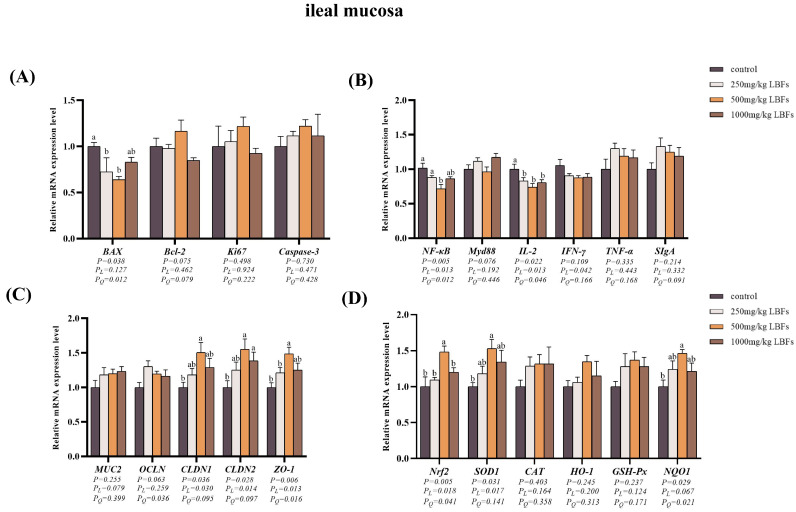
Effects of dietary LBFs on the mRNA expression of proliferation-, apoptosis-, immunity-, intestinal barrier function-, and antioxidant-related genes in the ileal mucosa. (**A**) Relative mRNA expression levels of proliferation- and apoptosis-related genes. (**B**) Relative mRNA expression levels of immune-related genes. (**C**) Relative mRNA expression levels of intestinal barrier-related genes. (**D**) Relative mRNA expression levels of antioxidant-related genes. Note: The mean and SEM are used to present the results, n = 6. Values with different superscript letters are significantly different, as shown by a, b (*p* < 0.05). *Q* and *L* are the quadratic and linear responses, respectively, to the levels of dietary supplementation with LBFs.

As depicted in Figure 2A and Figure 3A, compared with those of the control group, dietary supplementation with 250 and 500 mg/kg LBFs significantly decreased the relative mRNA expression of *BAX* in the ileal mucosa (*p* < 0.05), 500 mg/kg LBFs significantly decreased the relative mRNA expression of *BAX* in the jejunal mucosa and increased the relative mRNA expression of *Bcl-2* (*p* < 0.05), and no significant differences were observed in the relative mRNA expression levels of *Ki67* and *Caspase-3* among the groups (*p* > 0.05).

### 3.6. Immune-Related Gene Expression in Intestinal Mucosa

As illustrated in Figure 2B and Figure 3B, compared with the control group, dietary supplementation with 250, 500, and 1000 mg/kg LBFs significantly decreased the relative mRNA expression of *IL-2* in the jejunal and ileal mucosa (*p* < 0.05); dietary supplementation with 500 mg/kg of LBFs significantly decreased the relative mRNA expression of *NF-κB* in the jejunal and ileal mucosa and *TNF-α* in the jejunal mucosa (*p* < 0.05); and no significant differences were observed in the relative mRNA expression of *MyD88* and *IFN-γ* among the groups (*p* > 0.05).

### 3.7. Intestinal Barrier-Related Gene Expression in Intestinal Mucosa

As shown in Figure 2C and Figure 3C, compared with those of the control group, dietary supplementation with 500 and 1000 mg/kg LBFs significantly increased the relative mRNA expression of *OCLN* and *CLDN1* in the jejunal mucosa and *CLDN2* in the ileal mucosa (*p* < 0.05), and dietary supplementation with 500 mg/kg LBFs also significantly increased the relative mRNA expression of *CLDN2* and *ZO-1* in the jejunal mucosa and *CLDN1* and *ZO-1* in the ileal mucosa (*p* < 0.05).

### 3.8. Antioxidant-Related Gene and Protein Expression in Intestinal Mucosa

As depicted in Figure 2D and Figure 3D, compared with the control group, dietary supplementation with 500 and 1000 mg/kg LBFs significantly increased the relative mRNA expression of *Nrf2* and *GSH-Px* in the jejunal mucosa (*p* < 0.05), and dietary supplementation with 500 mg/kg LBFs significantly increased the relative mRNA expression of *NQO1* and *HO-1* in the jejunal mucosa, *Nrf2* and *SOD1* in the ileal mucosa, and *NQO1* in the ileal mucosa (*p* < 0.05).

**Figure 4:**
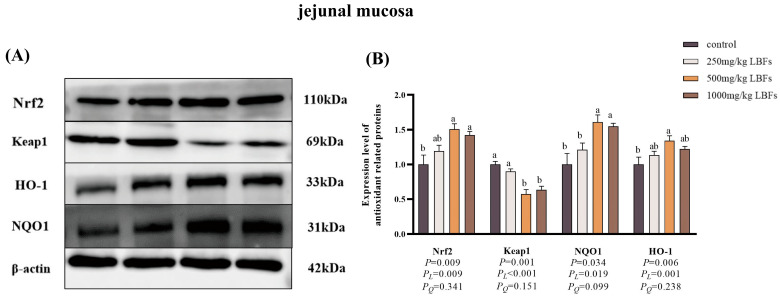
Effects of dietary LBFs on the expression of proteins related to the Nrf2 signaling pathway of jejunal mucosa in meat ducks (n = 6). Control: basal diet (control group); 250, 500, and 1000 mg/kg LBFs: basal diet further supplemented with 250, 500, and 1000 mg LBFs/kg, respectively. (**A**) Western blot of nuclear Nrf2, keap1, and β-actin proteins. (**B**) Relative expression of proteins Nrf2/β-actin and Keap1/β-actin. Values with different superscript letters are significantly different.

**Figure 5:**
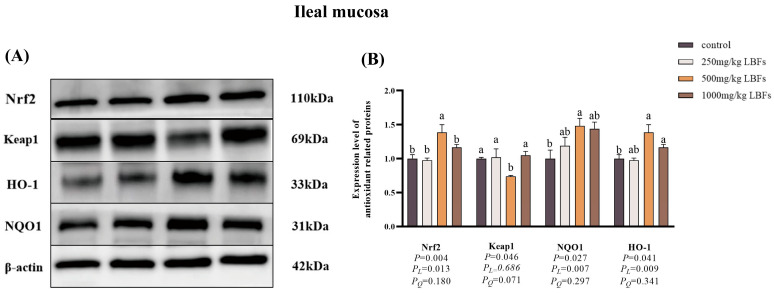
Effects of dietary LBFs on the expression of proteins related to the Nrf2 signaling pathway of the ileal mucosa in meat ducks (n = 6). Control: basal diet (control group); 250, 500, and 1000 mg/kg LBFs: basal diet further supplemented with 250, 500, and 1000 mg LBFs/kg, respectively. (**A**) Western blot of nuclear Nrf2, keap1, and β-actin proteins. (**B**) Relative expression of proteins Nrf2/β-actin and Keap1/β-actin. Values with different superscript letters are significantly different.

As illustrated in Figure 4 and Figure 5, compared with the control group, dietary supplementation with 500 and 1000 mg/kg LBFs significantly increased the relative protein expression of Nrf2 and HO-1 in the jejunal and ileal mucosa and significantly decreased the relative protein expression of Keap1 in the jejunal mucosa (*p* < 0.05), and dietary supplementation with 500 mg/kg LBFs significantly increased the relative protein expression of NQO1 in the jejunal and ileal mucosa and significantly decreased the relative protein expression of Keap1 in the ileal mucosa (*p* < 0.05).

## 4. Discussion

Owing to the global proliferation of regulations restricting antibiotic use in poultry feed, the use of plant extracts or phytochemicals in poultry nutrition to enhance growth performance and meat quality has become a new and important trend [17]. LB, traditionally used in China as a dual-purpose medicinal and food plant, contains bioactive compounds, particularly polyphenols and flavonoids, both of which have biological activities, such as antioxidant and immune enhancement. LBFs have substantial potential as plant-based additives to effectively replace antibiotics in the poultry industry; however, further research is required to determine their optimal dosage [14,18]. Therefore, we investigated the effects of incorporating 250, 500, and 1000 mg/kg LBFs into the diets of meat ducks.

Our results indicated that dietary supplementation with 250, 500, and 1000 mg/kg LBFs significantly increased BW and ADG during the entire experimental period. These findings are consistent with those of Abdel-Latif et al., who observed that dietary supplementation with 200, 400, and 800 mg/kg quercetin resulted in growth rate enhancements of 3.27%, 3.18%, and 2.32%, respectively, in broilers [19]. Moreover, Chen et al. found that dietary supplementation with 500 and 1000 mg/kg rutin significantly boosted BW and ADG in broilers [20]. Our study also revealed that, throughout the experimental period, meat ducks supplemented with 1000 mg/kg LBFs exhibited a significantly lower ADFI, those receiving 500 mg/kg LBFs showed a significantly higher ADFI, and ducks given 250 mg/kg LBFs did not demonstrate any significant change in ADFI. Therefore, we speculate that an appropriate amount of LBFs stimulated the ducks’ appetite and promoted food intake. The 250 mg/kg group exhibited an ADFI similar to the control, indicating that this dose did not significantly alter the ducks’ feeding behavior. In contrast, the 1000 mg/kg group showed a lower ADFI compared to the control, possibly due to the high concentration of LBFs exerting a slight inhibitory effect on feed intake. Regarding the FCR, improvements were observed across all dose groups, suggesting that LBF supplementation enhanced the ducks’ efficiency in utilizing feed.

Intestinal barrier permeability and integrity play critical roles in facilitating efficient digestion and absorption of nutrients. Impaired intestinal barrier function can lead to decreased animal production and increased health risks [21,22]. D-LA is a metabolic product generated during fermentation by gut microbiota that traverses the damaged barrier and enters the bloodstream [23]. LPS, the main constituent of the cell walls of Gram-negative bacteria, can enter the breached intestinal barrier and induce systemic inflammatory responses [24]. DAO is an enzyme present in intestinal epithelial cells that is released into the bloodstream from damaged intestinal epithelium, leading to an increase in serum DAO activity [25]. In this study, meat ducks fed diets supplemented with 500 mg/kg LBFs displayed lower serum levels of D-LA, LPS, and DAO activity than those of the control group, suggesting that daily supplementation with 500 and 1000 mg/kg LBFs can improve intestinal barrier function in meat ducks, which is consistent with the findings of Feng et al. in broiler chickens, in which dietary quercetin improved gut inflammation induced by LPS and enhanced gut function [26].

Furthermore, the intestinal barrier function is usually determined by the integrity of tight junction (TJ) complexes within the epithelial layer, owing to TJ complexes playing a critical role in preserving the apical intercellular spaces of epithelial cells lining the intestine [27]. Consequently, we assessed the expression of TJ-related genes in the jejunal and ileal mucosa. OCLN, CLDN-1, and CLDN-2 are transmembrane proteins integral to TJs that contribute to TJ integrity and regulate barrier function. ZO-1 is a scaffolding protein that links transmembrane proteins to the intracellular cytoskeleton [28]. Our research revealed that dietary supplementation with 500 mg/kg LBFs resulted in up-regulated mRNA expression of TJ-related genes, including *CLDN1*, *CLDN2*, and *ZO-1* in the jejunal and ileal mucosa of meat ducks, supporting existing studies that demonstrated that transepithelial electrical resistance (TER) in intestinal cells incubated with flavonoids increased significantly and that these changes in TER were due to the increased expression of TJ proteins, such as *ZO-1* and *ZO-2* [29]. In summary, our findings suggest that dietary supplementation with LBFs contributes to the regulation of intestinal barrier permeability and integrity, improving intestinal barrier function and growth performance.

Apoptosis is a genetically controlled, programmed cell death process that maintains homeostasis in a stable internal environment. This active process involves gene activation, expression, and regulation [30]. BAX is a pro-apoptotic gene belonging to the Bcl-2 family. When overexpressed in cells, BAX accelerates apoptosis in response to death signals [31,32]. Additionally, Bcl-2 is a well-known anti-apoptotic gene, also part of the Bcl-2 family [33]. Bcl-2 exerts its inhibitory effect on cell death by forming heterodimers with BAX and other pro-apoptotic family members, preventing the execution of apoptotic functions [34]. In our study, we observed that dietary supplementation of meat ducks with 500 mg/kg LBFs led to decreased *BAX* mRNA expression and increased *Bcl-2* mRNA expression in the jejunal and ileal mucosa, which supports the findings of Lee et al. that 3,4-Dihydroxyflavones had anti-apoptotic effects on etoposide-induced keratinocytes, reducing Caspase-3 protein expression in cells and inhibiting signaling pathways that promote apoptosis [35]. In summary, our findings suggest that dietary supplementation with LBFs improves the health status of intestinal mucosal cells in meat ducks by modulating the Bcl-2/BAX signaling pathway. This regulation could be one of the mechanisms by which the dietary addition of LBFs improves the intestinal morphology, nutrient absorption capacity, and growth performance of meat ducks.

In modern duck meat production, oxidative stress poses a significant threat to duck health and is characterized by an imbalance between oxidation and antioxidation processes within the body [36,37]. SOD can alleviate cellular oxidative damage by clearing free radicals, whereas GSH-Px and CAT, key peroxidases in vivo, enhance the body’s ability to decompose peroxide products [38,39]. The T-AOC serves as a comprehensive indicator of the overall status of the body’s antioxidant system [40]. The MDA content reflects the rate and intensity of lipid peroxidation in the body and the degree of oxidative damage to tissues [41]. In this study, compared with the control group, meat ducks fed a diet supplemented with 500 mg/kg LBFs demonstrated higher GSH-Px, CAT, T-SOD, and T-AOC activities in the jejunal and ileal mucosa and a lower MDA content. Studies have shown that flavonoids can inhibit the formation of MDA and enhance the activity of T-SOD and CAT, which is consistent with our results [42,43]. These findings suggest that dietary LBFs enhance the antioxidant capacity of meat ducks by strengthening the enzymatic and non-enzymatic antioxidant systems, alleviating oxidative stress. However, the enzymatic activity may differ across different segments of the intestine.

To further explore the molecular mechanism by which dietary LBFs improve intestinal antioxidant function in broilers, we assessed the mRNA expression of genes related to the Nrf2 signaling pathway and their corresponding protein expression. Nrf2, a pivotal transcription factor in oxidative stress response, regulates a series of antioxidant-related genes. When *Nrf2* gene expression is up-regulated, the transcription levels of downstream antioxidant genes, such as *NQO1* and *HO-1*, also increase, enhancing the overall antioxidant capacity of the organism [44,45]. The results of this study indicated that in meat ducks fed diets containing 500 or 1000 mg/kg LBFs, the relative mRNA expression levels of *Nrf2*, *HO-1*, *NQO1*, *GSH-px*, and *SOD1* were up-regulated in the jejunal and ileal mucosa, consistent with the findings of Abdel in broiler chickens fed diets supplemented with 400 or 800 mg/kg quercetin, which led to increased *SOD1* and *GSH-Px* mRNA expression [19]. In our study, Western blotting results showed that dietary supplementation with 500 mg/kg LBFs in meat ducks was associated with higher Nrf2, HO-1, and NQO1 protein expression and lower Keap1 protein expression in the jejunal and ileal mucosa than for the control group, consistent with the up-regulated mRNA expression of *Nrf2*, *HO-1*, and *NQO1*. Studies have found that after rutin pretreatment, rats exhibited reduced liver MDA content, elevated CAT activity, and increased Nrf2 and HO-1 protein expression, which corroborates our findings in this study [46]. In summary, dietary LBFs can enhance the antioxidant capacity of meat duck intestines and mitigate oxidative stress by activating the Nrf2 signaling pathway.

Frequent inflammatory reactions in poultry can weaken the effectiveness of the immune system, reduce the effectiveness of vaccinations, and slow the natural immune response [47]. Therefore, in this study, we evaluate the effects of dietary supplementation with different concentrations of LBFs on the expression of immune- and inflammation-related genes in meat ducks. The results indicated that dietary supplementation with 500 mg/kg LBFs decreased *NF-κB*, *IL-2* mRNA expression in the jejunal and ileal mucosa and *TNF-α* mRNA expression in the jejunal mucosa compared with those in the control. NF-κB is a crucial transcription factor that regulates the expression of immune-related genes and pro-inflammatory factors such as IL-2 and TNF-α [48]. TNF-α is a pleiotropic cytokine that plays an essential role in inflammation, apoptosis, and immune system development by promoting proliferation and differentiation of immune cells [49]; IL-2 is a cytokine that determines the differentiation and growth status of T cells and enhances their cytotoxic activity [50]. A study revealed that pretreatment with luteolin (rich in flavonoids) inhibited NF-κB signal transduction and subsequent pro-inflammatory gene expression in murine distal intestinal epithelial cells induced by LPS, which is consistent with our findings [51]. Reactive oxygen species produced under oxidative stress conditions effectively activate the NF-κB signaling pathway and up-regulate the expression of pro-inflammatory factors in meat ducks. Existing studies have found that Nrf2 and HO-1 can negatively regulate the expression of genes involved in inflammatory reactions through antagonizing transcription factors like NF-κB [48,52]. Therefore, activation of the Nrf2 signaling pathway and inhibition of the NF-κB signaling pathway may represent the mechanism by which dietary LBFs improve the immune response in meat ducks.

## 5. Conclusions

In summary, dietary supplementation with LBFs can enhance intestinal barrier function by reducing gut permeability and up-regulating the expression of genes associated with TJ, improving growth performance, immunity, and antioxidant functions in meat ducks. This beneficial effect is probably closely related to the activation of the Nrf2 signaling pathway and suppression of the NF-κB signaling pathway. Considering the comprehensive benefits, the optimal dietary inclusion level of LBFs in ducks was 500 mg/kg.

## Figures and Tables

**Table 1:** Ingredient and nutrient levels of the basal diets (fed basis).

Item	Starter Period (1~21 d)	Grower Period (22~42 d)
Ingredient (%)		
Corn	66.7	68.98
Soybean meal	20.4	20.1
Rice bran	4.33	4.09
Corn gluten meal	3.70	1.70
Dicalcium phosphate	1.87	1.98
Limestone	1.08	1.07
L-Lysine	0.32	0.42
DL-Methionine	0.30	0.36
Sodium chloride	0.30	0.30
Premix ^A^	1	1
Total	100	100
Nutrient ^B^		
ME (MJ·kg^−1^) ^C^	12.48	12.39
Crude protein (%)	21.79	18.15
Lysine (%)	1.39	1.25
Methionine (%)	0.68	0.65
Calcium (%)	0.96	1.00
Total phosphorus (%)	0.67	0.72

^A^ Premix provided per kilogram of diet: vitamin A 10,000 IU, vitamin D_3_ 2000 IU, vitamin E 20 mg, vitamin K 0.5 mg, nicotinic acid 60 mg, calcium pantothenate 11 mg, pyridoxine 2.5 mg, riboflavin 4.0 mg, biotin 0.2 mg, folic acid 0.6 mg, thiamine 3 mg, Cu (provided as copper sulfate) 8 mg, Fe (provided as ferrous sulfate) 80 mg, Mn (provided as manganese oxide) 80 mg, Zn (provided as zinc oxide) 60 mg, Se (provided as sodium selenite) 0.20 mg. ^B^ Nutrient levels were calculated values. ^C^ ME: metabolizable energy.

**Table 2:** Sequences used for real-time PCR primers.

Gene ^A^	Accession No.	Primers Sequence (5′→3′)	Products Size
*β-actin*	NM_205518.1	F: TGCTGTGTTCCCATCTATCG	150
		R: TTGGTGACAATACCGTGTTCA	
*Nrf2*	XM_027460922.2	F:CAGCTCAGCGCATTCAGTCA	165
		R:ATGCAGCTGAAGAAGCCTCA	
*NQO1*	XM_027466610.2	F:GATCTGATCATCTTCCAGTTCCCA	161
		R:GTGGTGAATGACAGCATGGC	
*SOD1*	XM_027449207.2	F:TCGGCAACGTGACTGCTAAA	163
		R:TTCCCAGTTAGCGTGCTCTC	
*GSH-Px*	KU_048803.1	F:CAGTACATCATCTGGTCGCC	127
		R:CCTGGATCTTGATGGTTTCG	
*HO-1*	KU_048806.1	F:CCCATGCCTACACTCGCTAT	217
		R:GCCTCCTCCAAGACTCGTTT	
*CAT*	KU_048802.1	F:CTGTTGAGGAAGCAGGAAGG	101
		R:GAAAGACCAGGATGGGTAGTTG	
*TNF-α*	XM_027471963.2	F:CTCACGGACAAGGAAGGTTGG	135
		R:GGCTTCTGCCATCAGCTCTT	
*Myd88*	NM_001310832.1	F:AGCTTATAGAAAGGAGGTGTCGG	131
		R:AATCAGCCGCTTGAGACGAG	
*IL-2*	NM_001310373.1	F:AGTGCAGCTGGCAAACTCTG	156
		R:TTCCTGGGGGAATTAGGTCCATA	
*IFN-γ*	NM_001310417.1	F:CAGGTCCACGAGGTCTTT	146
		R:TGAGCCAGATTGTTTCCC	
*NF-κB*	XM_027465251.2	F:TTCATGGGATGCAGATACGGC	101
		R:CAAGGGACGAGCTCGAATGT	
*SlgA*	U27222.1	F:TCGCTCAAGGAACCCATCGT	174
		R:GCGGGACCACGAGAACTTCA	
*MUC2*	XM_005024513.2	F:GGGCGCTCAATTCAACATAAGTA	150
		R:TAAACTGATGGCTTCTTATGCGG	
*CLDN1*	XM_013108556.1	F:TCATGGTATGGCAACAGAGTGG	146
		R:CGGGTGGGTGGATAGGAAGT	
*CLDN2*	XM_005009661.2	F:CTCCTCCTTGTTCACCCTCATC	160
		R:GAACTCGCTCTTGGGTTTGTG	
*OCLN*	XM_013109403.1	F:GGCTTCCTCATCGTCCTCTTG	160
		R:TCTCGTACTGCGACTCGTCCAC	
*ZO-1*	XM_013104936.1	F:ACGCTGGTGAAATCAAGGAAGAA	255
		R:AGGGACATTCAACAGCGTGGC	
*BAX*	XM_013106199.3	F:AAGGCCTGCCTTGCTTTTGA	127
		R:CAGTGCTTCCAGCAGGGTAAAT	
*Bcl-2*	XM_005028719.1	F:ACCTGGTTCTGAATAAGTGGGAT	187
		R:GGTTGTCTTCTCAGTGTTGCCT	
*Ki67*	XM_038180817.1	F:CCTCTGAAGCACGGAGATGT	132
		R:CTGAACATGAAGAACCTGCCG	
*Caspase3*	XM_021279218.3	F:TTGTCAGCCTCGCAGTTGAT	198
		R:ACACACTCTCCCATCTCTGGA	

^A^ *BAX*: B-cell lymphoma 2-associated X; *Bcl-2*: B-cell lymphoma 2; *CLDN1*: claudin-1; *CLDN2*: claudin-2, *GSH-Px*: glutathione peroxidase; *HO-1*: heme oxygenase-1; *IL-2*: interleukin-2; *IFN-γ*: interferon-c; *MUC2*: mucin 2; *MyD88*: myeloid differentiation factor 88; *NF-κB*: nuclear factor kappa-B; *NQO1*: NAD(P)H quinone oxidoreductase 1; *Nrf2*: nuclear factor erythroid-2-related factor 2; *OCLN*: occludin; *SOD1*: superoxide dismutase1; *TNF-α*: tumor necrosis factor-a; *ZO-1*: zonula occludens-1, *CAT*: catalase, *SlgA*: Secretory Immunoglobulin A; *Ki67*: marker of proliferation Ki-67; *Caspase3*: cysteinyl aspartate-specific protease 3.

**Table 3:** Effects of dietary LBFs on growth performance of broiler ducks.

Item ^A^	Dietary Treatments ^B^				SEM ^C^	*p* Value ^D^		
	0	250	500	1000		*p*	*L*	*Q*
Body weight								
1 d (g)	51.42	51.50	51.33	51.50	0.224	0.994	0.969	0.932
21 d (kg)	1.11	1.12	1.12	1.16	0.007	0.143	0.066	0.244
42 d (kg)	2.69 ^c^	2.77 ^b^	2.84 ^ab^	2.86 ^a^	0.023	0.001	<0.001	0.222
ADG, g/d								
1~21 d	50.61	50.92	50.69	52.55	0.342	0.142	0.065	0.243
21~42 d	74.80 ^b^	78.45 ^ab^	82.30 ^a^	81.03 ^a^	0.863	0.004	0.001	0.080
1~42 d	62.70 ^c^	64.69 ^b^	66.49 ^ab^	66.79 ^a^	0.463	0.001	<0.001	0.222
ADFI, g/d								
1~21 d	81.34 ^a^	76.98 ^b^	78.49 ^b^	77.07 ^b^	0.568	0.011	0.013	0.130
21~42 d	172.88 ^b^	169.36 ^bc^	185.49 ^a^	161.65 ^c^	2.418	0.001	0.271	0.008
1~42 d	127.11 ^b^	123.17 ^bc^	131.99 ^a^	119.36 ^c^	1.244	<0.001	<0.001	0.0016
FCR								
1~21 d	1.61 ^a^	1.51 ^b^	1.55 ^ab^	1.47 ^b^	0.017	0.017	0.007	0.825
21~42 d	2.31 ^a^	2.16 ^ab^	2.26 ^a^	1.99 ^b^	0.037	0.006	0.004	0.369
1~42 d	2.02 ^a^	1.91 ^bc^	1.98 ^b^	1.79 ^c^	0.025	<0.001	0.001	0.277

^A^ ADFI: average daily feed intake; ADG: average daily gain; FCR: feed conversion ratio. ^B^ 0 (control), 250, 500, and 1000: basal diet further supplemented with 0 (control group), 250, 500, and 1000 mg/kg LBFs, respectively. ^C^ SEM: pooled standard error of means. ^D^ *L*: linear; *Q*: quadratic. Orthogonal polynomials were used to evaluate linear and quadratic responses to the levels of dietary LBFs (n = 6). ^a–c^ Means in the same row with no common superscript differ significantly (*p* < 0.05).

**Table 4:** Effects of dietary LBFs on organ indices of broiler ducks (day 42, g/kg).

Item	Dietary Treatments ^A^				SEM ^B^	*p* Value ^C^		
	0	250	500	1000		*p*	*L*	*Q*
Pectoral muscles	85.67	102.70	90.63	87.77	3.043	0.197	0.826	0.103
Liver	25.37	24.51	24.43	23.37	0.488	0.706	0.266	0.966
Pancreas	2.51	2.27	2.03	1.90	0.103	0.161	0.028	0.779
Spleen	1.03	0.75	0.86	0.78	0.106	0.820	0.529	0.668
Bursa	1.16	1.08	1.03	1.04	0.040	0.677	0.278	0.600
Abdominal fat	11.42	11.81	13.03	10.92	0.502	0.520	0.956	0.235

^A^ 0 (control), 250, 500 and 1000: basal diet further supplemented with 0 (control group), 250, 500 and 1000 mg/kg LBFs, respectively. ^B^ SEM: pooled standard error of means. ^C^ *L*: linear; *Q*: quadratic. Orthogonal polynomials were used to evaluate linear and quadratic responses to the levels of dietary LBFs (n = 6).

**Table 5:** Effects of dietary LBFs on antioxidant capacity of jejunal mucosa of broiler ducks (day 42).

Item ^A^	Dietary Treatments ^B^				SEM ^C^	*p* Value ^D^		
	0	250	500	1000		*p*	*L*	*Q*
GSH (umoL/gprot)	288.71	296.40	298.88	291.04	3.280	0.702	0.418	0.992
CAT (U/mgprot)	20.01 ^b^	20.07 ^b^	22.42 ^a^	21.26 ^ab^	0.328	0.014	0.016	0.389
T-AOC (U/mgprot)	1.34 ^b^	1.42 ^ab^	1.51 ^a^	1.46 ^a^	0.019	0.010	0.006	0.048
T-SOD (U/mgprot)	19.26	19.80	20.87	20.39	0.618	0.831	0.975	0.983
MDA (nmoL/mgprot)	2.64	1.96	1.60	1.95	0.177	0.209	0.126	0.141
GSH-Px (U/mgprot)	90.10 ^b^	95.29 ^ab^	100.85 ^a^	100.31 ^a^	1.352	0.007	0.001	0.197

^A^ MDA: malondialdehyde; T-SOD: total superoxide dismutase; CAT: catalase; GSH: glutathione, GSH-Px: glutathione peroxidase; T-AOC: total antioxidant capacity. ^B^ 0 (Control), 250, 500, and 1000: basal diet further supplemented with 0 (control group), 250, 500, and 1000 mg/kg LBFs, respectively. ^C^ SEM: pooled standard error of means. ^D^ *L*: linear; *Q*: quadratic. Orthogonal polynomials were used to evaluate linear and quadratic responses to the levels of dietary LBFs (n = 6). ^a,b^ Means in the same row with no common superscript differ significantly (*p* < 0.05).

**Table 6:** Effects of dietary LBFs on antioxidant capacity of ileal mucosa of broiler ducks (day 42).

Item ^A^	Dietary Treatments ^B^				SEM ^C^	*p* Value ^D^		
	0	250	500	1000		*p*	*L*	*Q*
GSH (umol/gprot)	191.75 ^b^	237.94 ^a^	252.05 ^a^	230.51 ^a^	6.327	0.001	0.005	0.001
CAT (U/mgprot)	19.85	20.58	20.76	20.66	0.176	0.252	0.104	0.237
T-AOC (U/mgprot)	1.33 ^c^	1.39 ^bc^	1.49 ^a^	1.44 ^ab^	0.019	0.010	0.021	0.009
T-SOD (U/mgprot)	14.12 ^b^	16.36 ^ab^	17.63 ^a^	17.16 ^a^	0.511	0.061	0.028	0.344
MDA (nmol/mgprot)	1.54 ^b^	1.41 ^ab^	1.18 ^a^	1.16 ^a^	0.545	0.021	0.003	0.556
GSH-Px (U/mgprot)	79.71	82.16	85.05	84.86	1.131	0.302	0.220	0.558

^A^ MDA: malondialdehyde; T-SOD: total superoxide dismutase; CAT: catalase; GSH: glutathione, GSH-Px: glutathione peroxidase; T-AOC: total antioxidant capacity. ^B^ 0 (Control), 250, 500, and 1000: basal diet further supplemented with 0 (control group), 250, 500, and 1000 mg/kg LBFs, respectively. ^C^ SEM: pooled standard error of means. ^D^ *L*: linear; *Q*: quadratic. Orthogonal polynomials were used to evaluate linear and quadratic responses to the levels of dietary LBFs (n = 6). ^a–c^ Means in the same row with no common superscript differ significantly (*p* < 0.05).

## Data Availability

The original contributions presented in the study are included in the article; further inquiries can be directed to the corresponding author.
